# Increase in antibiotic resistance in diabetic foot infections among peruvian patients: a single-center cross-sectional study

**DOI:** 10.3389/fendo.2023.1267699

**Published:** 2023-12-05

**Authors:** Jeel Moya-Salazar, Jackelina M. Chamana, Daniela Porras-Rivera, Eliane A. Goicochea-Palomino, Carmen R. Salazar, Hans Contreras-Pulache

**Affiliations:** ^1^ Faculties of Health Science, Universidad Privada del Norte, Lima, Peru; ^2^ Digital Transformation Center, Universidad Norbert Wiener, Lima, Peru; ^3^ School of Medicine, Universidad Pedagógica y Tecnológica de Colombia, Tunja, Colombia; ^4^ School of Medical Technologist, Faculties of Health Science, Universidad Tecnológica del Perú, Lima, Peru; ^5^ Infectious Unit, Nesh Hubbs, Lima, Peru

**Keywords:** diabetic foot, infections, staphylococcus aureus, antibiotic resistance, *Escherichia coli*, diabetes mellitus

## Abstract

**Background:**

Diabetic foot is one of the most significant complications in individuals with diabetes and is closely associated with lower limb amputation. The antibiotic susceptibility patterns of these bacterial isolates play a critical role in guiding effective treatment strategies We aimed to determine the most common bacterial agents causing diabetic foot infections in a tertiary-care hospital in Peru.

**Methods:**

Clinical and microbiological data were collected from 181 patients diagnosed with diabetic foot infections and positive microbiological culture results. All the samples were analyzed with the Vitek 2 compact system and the cut-off points were defined with the CLSI M100 guide. The data were segregated based on mono-microbial or poly-microbial cultures, bacterial types, and antibiotic susceptibility profiles.

**Results:**

A total of 32 bacterial species were identified, predominantly Gram-negative (63%). The most frequent bacterial agents isolated were *Staphylococcus aureus* (19.9%), *Escherichia coli* (12.2%), *Pseudomonas aeruginosa* (8.3%), and *Proteus vulgaris* (6.6%). These bacteria commonly exhibited resistance to Ampicillin, Ciprofloxacin, Levofloxacin, Trimethoprim-sulfamethoxazole, and Cefuroxime. *E. coli* showed the highest antibiotic resistance (19 antibiotics), while Gentamicin, Tobramycin, and Levofloxacin demonstrated the highest sensitivity against the most prevalent bacteria. Gram-negative bacteria also exhibited notable antibiotic-susceptibility to Meropenem, Piperacillin/tazobactam, and Amikacin. Regarding the presence of Extended-Spectrum Beta-Lactamase, 54 isolates tested positive, with 35 (64.8%) and 14 (42.4%) of these being *S. aureus and E. coli*.

**Conclusions:**

Bacterial agents causing diabetic foot infections pose a constant concern, particularly due to the increasing antibiotic resistance observed. This difficulty in treating the condition contributes to a higher risk of amputation and mortality. Further research on bacterial susceptibility is necessary to determine appropriate dosages for pharmacological treatment and to prevent the overuse of antibiotics.

## Graphical Abstract

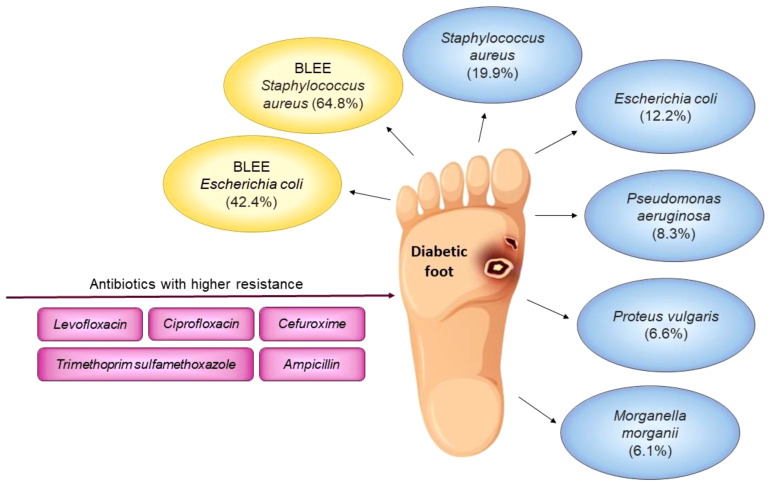

## 1 Introduction

Globally, diabetes mellitus is a widespread health concern, affecting more than 529 million individuals, with prevalence spanning across all age groups, from 65 to 95 years (1). This surge in the number of diabetes cases can be attributed to the escalating risk factors, including excess body weight, obesity, sedentary lifestyles, and imbalanced diets (2). Diabetes has a significant impact on the quality of life and reduces life expectancy of affected individuals. Moreover, it gives rise to various morbidity issues, encompassing both microvascular and macrovascular complications. These complications manifest as visual impairments, ranging from partial loss of vision to complete blindness, as well as serious health conditions such as acute myocardial infarction, renal failure, stroke, and peripheral neuropathy and peripheral arterial diseases that may necessitate amputations (1).

Diabetic foot is a significant complication of Diabetes Mellitus, often associated with diabetic sensory-motor polyneuropathy (in fact, diabetic polyneuropathy alone accounts for 50% of diabetic foot cases), occlusive peripheral arterial disease, or a combination of both (3). These patients have an increased risk of infection, affecting approximately 50% of them, and infection is the leading factor associated with lower limb amputations (4). Infections in these patients usually result from skin discontinuity caused by trauma (mechanical/thermal) or ulceration. Diabetic foot infection is defined as an infection in soft tissue or bone anywhere below the malleolus in a diabetic individual (5).

The main microorganisms isolated in diabetic foot infections are Staphylococcus aureus, Proteus spp, Escherichia coli, *Peptostreptococcus, Veillonella*, and *Bacteroides* (3). However, microbiology of the lesions can vary based on different patient factors, including the characteristics and duration of the lesion, prior use of antibiotics, and local microbiology (6). Generally, most infections are polymicrobial, hence the use of empiric broad-spectrum antibiotics are necessary initially and then tailoring treatment based on antibiotic susceptibility results. In severe infections, surgical debridement may also be required (7).

In Peru, the two main complications of diabetes are diabetic foot and peripheral diabetic neuropathy, with a prevalence of 30% and 7%, respectively (8). Regarding the local microbiology of diabetic foot infections, it varies among different populations, ranging from gram-positive isolates such as Staphylococcus aureus (9), to bacteria like Escherichia coli and Enterococcus faecalis (10), which show high resistance to at least five commonly used antibiotics, such as ciprofloxacin, levofloxacin, nitrofurantoin, trimethoprim/sulfamethoxazole, and ampicillin/sulbactam (9). Despite these previous reports, not all hospitals have characterized bacterial infections in diabetic foot patients, making it important to understand changes in pathogen frequency and potential resistance patterns to avoid complications and ensure proper microbiological surveillance.

The objective of this study was to determine the most frequent bacterial agents causing diabetic foot infections in a tertiary hospital in Peru. This study also aimed to characterize the antibiotic resistance profile of the infectious isolates from diabetic foot infections, highlighting differences between nosocomial and community-acquired pathogens.

## 2 Methods

### 2.1 Study design and setting

This retrospective study was conducted at the María Auxiliadora Hospital, a tertiary hospital located in the district of San Juan de Miraflores, Lima (Peru). Managed by the Ministry of Health, this hospital facility in the southern region proudly offers an extensive capacity of around 472 beds and accommodates approximately two thousand daily consultations. Around a thousand consultations per month are about type 1 and 2 diabetes in all care departments. It serves as an essential healthcare institution for the local community, playing a crucial role in meeting their medical needs.

### 2.2 Population and inclusion criteria

The study population consisted of 181 patients with type two diabetes mellitus and diagnosed with diabetic foot (11). The clinical and microbiological data of these patients were considered as the unit of analysis based on the following inclusion criteria:

- Tissue samples from diabetic foot, whether from the hospitalization area, emergency department, or the diabetic foot unit in the outpatient consultation.
- Samples with microbiological cultures containing Gram-positive and Gram-negative bacterial isolates.
- Samples with complete information on susceptibility profiles.

Patients with incomplete data records, cultures with non-bacterial isolates, samples from other areas different from the foot of a diabetes patient, patients with type 1 diabetes and gestational diabetes, and samples from patients with foot or lower limb amputations not related to diabetes were excluded.

### 2.3 Microbiological and clinical data gathering

All samples were analyzed using the Vitek 2 compact system (bioMérieux, LePort, France) following standardized operational procedures of the hospital. Data were directly collected from the system (clinical isolation data and antibiotic susceptibility profile) into a data collection form for the study, which also included clinical information (demographic and symptoms data) obtained from the SIGHOS system (12). SIGHOS, the System for Integrated Health Information Management, is a robust clinical data system within the Comprehensive Health Insurance (SIS) framework under the Ministries of Health. Its primary function is to seamlessly connect and integrate health care network data, facilitating both epidemiological analysis and clinical monitoring. The data were categorized according to mono-microbial or poly-microbial cultures, bacterial types, susceptibility profiles, patient age, and gender.

### 2.4 Statistical analysis and ethical considerations

Data analysis was performed using SPSS v24.0 (IBM, Armonk, US) for Windows. Descriptive analysis was used to estimate the frequency of each bacterial isolation. The clinical data obtained from SIGHOS were analyzed descriptively. Antimicrobial resistance categories (sensitive, intermediate, and resistant) were defined based on the Vitek 2 compact cut-off and CLSI M100 guidelines (13). Additionally, the frequency of mono or poly-microbial cultures and extended-spectrum beta-lactamase (ESBL) presence were identified.

This study has adhered to the guidelines of the Declaration of Helsinki (14). It has also received approval from the Ethics Committee of the Hospital María Auxiliadora (HMA/CIEI/008/2021, May 26, 2021) and the Universidad Norbert Wiener (Exp.528-2021, April 26, 2021).

## 3 Results

Out of a total of 181 positive cultures obtained from diabetic foot samples collected between January and December 2019, 128 were from male patients (70.7%), and an equal number were from the outpatient clinic area (128, 70.7%). Most patients belonged to the age group of 61 to 70 years, accounting for 35.9% (65/181), followed by 51 to 60 with 30.9% (56/181) and 71 to 80 years with 14.3% (26/181). In smaller proportions, there were groups aged 41 to 50 years with 8.8% (16/181) and >40 years with 1.7% (3/181).

A total of 32 bacterial species were identified, with 21 being Gram-negative (63%) and 11 Gram-positive (37%). The most frequently isolated Gram-positive species was Staphylococcus aureus with 36 isolations (19.9%), followed by Enterococcus faecalis with 9 isolations (5.0%). Among the Gram-negative bacteria, Escherichia coli had the highest frequency with 22 isolations (12.2%), followed by Pseudomonas aeruginosa with 15 isolations (8.3%) and Proteus vulgaris with 12 isolations (6.6%) (**Table 1**).

**Table 1:** Frequency of bacterial isolations in diabetic foot.

Isolated bacteria		n	%
**Gram negative bacteria**		114	63.0%
* Acinetobacter baumannii complex/haemolyticus*		6	3.3%
* Burkholderia cepacia complex*		1	0.6%
* Citrobacter sp*		4	2.2%
* Citrobacter freundii*		2	1.1%
* Citrobacter murliniae*		1	0.55%
* Citrobacter youngae*		1	0.55%
* Enterobacter sp*		13	7.2%
* Enterobacter cloacae*		9	5.0%
* Enterobacter aerogenes*		2	1.1%
* Enterobacter cancerogenus*		1	0.55%
* Enterobacter hormaechei*		1	0.55%
* Escherichia coli*		22	12.2%
* Klebsiella sp*		11	6.1%
* Klebsiella oxytoca*		1	0.6%
* Klebsiella pneumoniae*		10	5.5%
* Morganella morganii*		11	6.1%
* Proteus sp*		19	10.5%
* Proteus mirabilis*		7	3.9%
* Proteus vulgaris*		12	6.6%
* Providencia rettgeri*		4	2.2%
* Pseudomona sp*		16	8.8%
* Pseudomonas aeruginosa*		15	8.3%
* Pseudomonas stutzeri*		1	0.5%
* Serratia sp*		3	1.7%
* Serratia fonticola*		1	0.6%
* Serratia marcescens*		2	1.1%
* Stenotrophomonas maltophilia*		4	2.2%
**Gram-positive bacteria**		67	37.0%
* Enterococcus sp*		10	5.5%
* Enterococcus faecalis I’ll*		9	5.0%
* Enterococcus faecium*		1	0.5%
* Staphylococcus sp*		55	30.4%
* Staphylococcus aureus*		36	19.9%
* Staphylococcus cohnii subsp. Cohnii*		1	0.55%
* Staphylococcus epidermidis*		3	1.7%
* Staphylococcus haemolyticus*		8	4.4%
* Staphylococcus hominis subesp. Hominis*		1	0.55%
* Staphylococcus schleiferi subespecie coagulans*		1	0.55%
* Staphylococcus sciuri*		3	1.7%
* Staphylococcus xylosus*		2	1.1%
* Streptococcus dysgalactiae subspecies dysgalacti*		2	1.1%
TOTAL		181	100%

**Figure 1:**
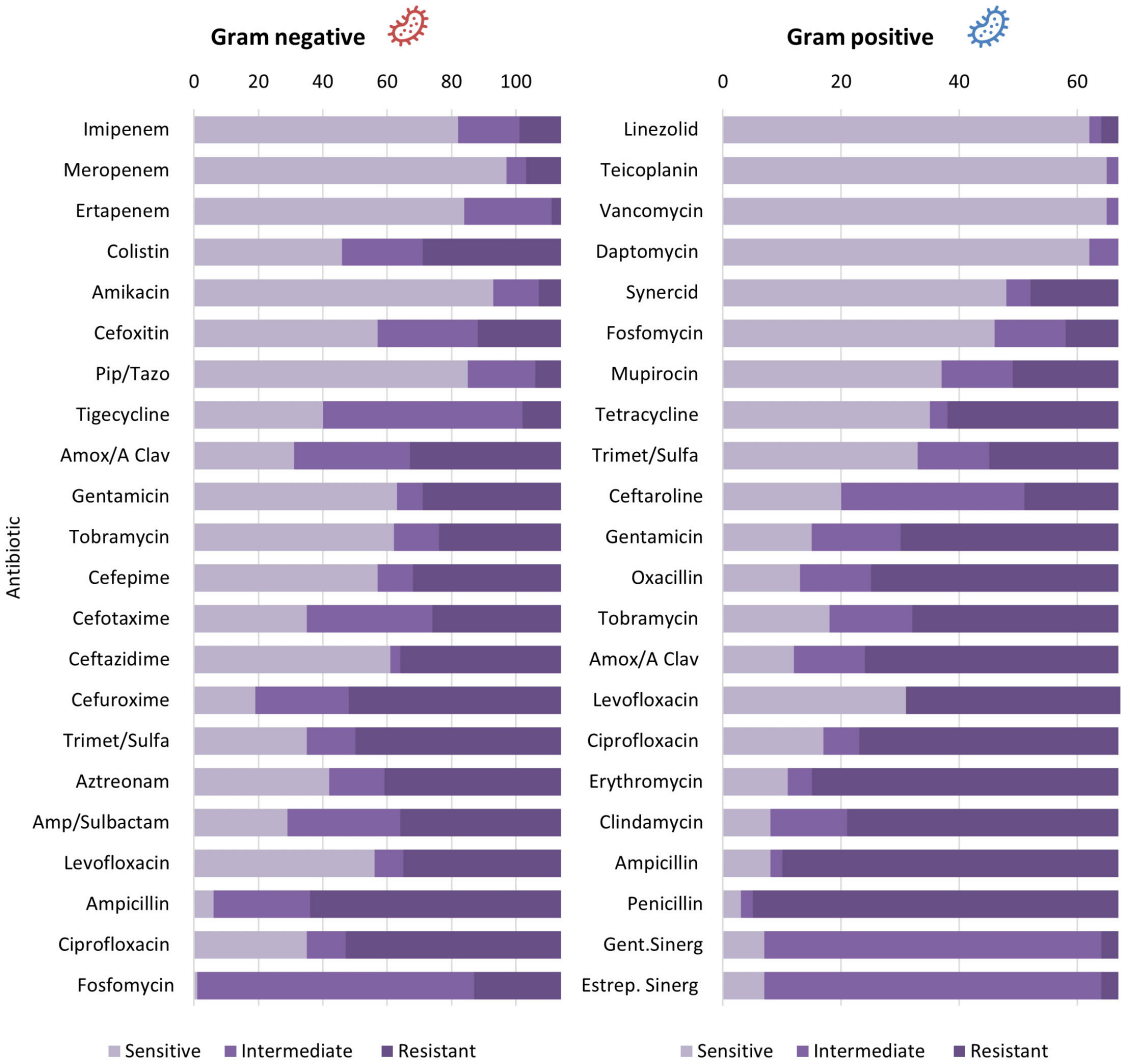
Antibiotic susceptibility of Gram positive and negative bacterial isolates in diabetic foot.

Regarding the susceptibility pattern, among the 114 isolates of Gram-negative bacteria, the antibiotics with the highest antimicrobial resistance were Ampicillin (89.7%), Cefuroxime (75.9%), followed by Trimethoprim-sulfamethoxazole (64.6%), and Ciprofloxacin (61.5%); the lowest resistance was observed for Ertapenem with 3.4%. On the other hand, the highest antimicrobial sensitivity was observed for Carbapenems (> 85.4%), Amikacin (85.3%), followed by Piperacillin/tazobactam (82.5%), while the lowest sensitivity was observed for Ampicillin (6.9%) and Fosfomycin (1.5%). Among the 67 Gram-positive bacteria, the antibiotics with the highest antimicrobial resistance were Penicillin (95.4%), Ampicillin (87.7%), Clindamycin (80.7%), and Oxacillin (76.4%). Daptomycin, Vancomycin, and Teicoplanin were resistant in all isolates, while Penicillin (4.6%) showed the lowest resistance (**Figure 1**).

In terms of frequencies, the order of bacteria with the highest isolation rates was *Staphylococcus aureus* (19.9%), *Escherichia coli* (12.2%), *Pseudomonas aeruginosa* (8.3%), *Proteus vulgaris* (6.6%), and *Morganella morganii* (6.1%). While Gram-negative bacteria had the highest number of isolations, *Staphylococcus* aureus was the most frequently isolated species.

Considering the bacteria with the highest incidence *(Staphylococcus aureus, Escherichia coli, Pseudomonas aeruginosa, Proteus vulgaris, Morganella morganii*), it was observed that they share a higher percentage of resistance to Ampicillin, Ciprofloxacin, Levofloxacin, Trimethoprim-sulfamethoxazole, and Cefuroxime. *Escherichia coli* showed the highest number of antibiotic resistance (19 antibiotics), followed by *Proteus vulgaris* (17 antibiotics) (**Table 2**).

**Table 2:** Antibiotic resistance of the major bacterial isolates in diabetic foot.

	*Staphylococcus aureus (n=36)*	*Escherichia coli (n=22)*	*Pseudomonas aeruginosa (n=15)*	*Proteus vulgaris (n=12)*	*Morganella morganii (n=11)*
Antibiotic	R (%)	R (%)	R (%)	R (%)	R (%)
Imipenem	_	-	47	-	9
Meropenem	_	-	47	-	-
Ertapenem	_	5	_	-	-
Colistin	_	5	20	92	100
Amikacin	_	-	27	-	-
Cefoxitin	_	14	_	8	18
Pip/Tazo	_	14	20	-	-
Tigecycline	_	15	_	33	-
Amox/A Clav	69	36	_	25	100
Gentamicin	61	68	53	17	9
Tobramycin	61	55	40	17	18
Cefepime	_	68	47	25	9
Cefotaxime	_	73	_	42	_
Ceftazidime	_	73	33	33	18
Cefuroxime	_	77	_	92	100
Trimet/Sulfa	31	77	_	75	82
Aztreonam	_	77	73	50	18
Amp/Sulbactam	_	68	_	25	100
Levofloxacin	67	82	47	8	36
Ampicillin	97	90	_	92	100
Ciprofloxacin	67	90	60	50	73
Fosfomycin	17	18	_	25	91
Ceftaroline	44	_	_	_	_
Clindamycin	78	_	_	_	_
Daptomycin	-	_	_	_	_
Erythromycin	75	_	_	_	_
Linezolid	-	_	_	_	_
Mupirocin	19	_	_	_	_
Oxacillin	67	_	_	_	_
Penicillin	97	_	_	_	_
Synercid	14	_	_	_	_
Teicoplanin	-	_	_	_	_
Tetracycline	22	_	_	_	_
Vancomycin	-	_	_	_	_

- means that no isolates with resistance to that antibiotic were found.

The antibiotics that showed the highest sensitivity against the most incident bacteria were Gentamicin (46.9%, 45/96), Tobramycin (46.9%, 45/96), and Levofloxacin (34.4%, 33/96). Notably, Meropenem (86.6%, 52/60), Piperacillin/tazobactam (81.6%, 49/60), and Amikacin (83.3%, 50/60) showed significant sensitivity against Gram-negative bacteria.

**Figure 2:**
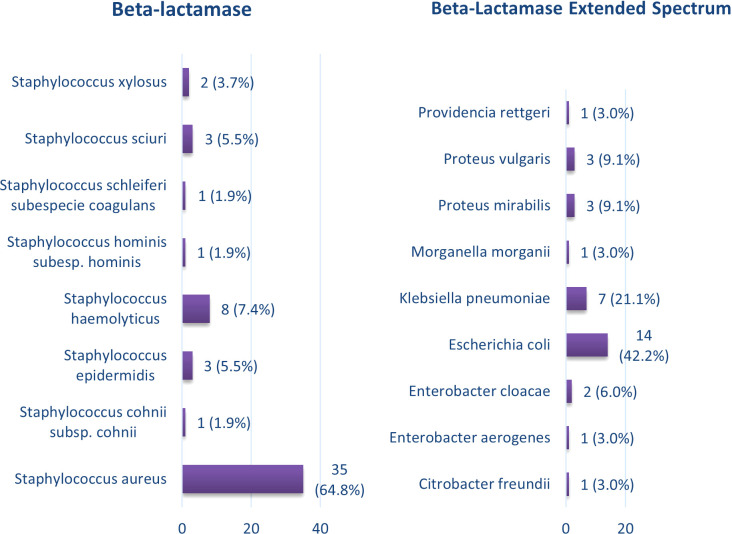
Bacterial isolates in diabetic foot with the presence of extended-spectrum beta-lactamases.

Regarding the presence of Extended-Spectrum Beta-Lactamase (ESBL), 54 isolates tested positive, with 35 (64.8%) of these being *Staphylococcus aureus*. *Staphylococcus haemolyticus* followed with 8 positive cases (7.4%). Additionally, a total of 33 isolates were related to Extended-Spectrum Beta-Lactamase, with *Escherichia coli* showing the highest positivity at 14 (42.4%), followed by *Klebsiella pneumoniae* with 7 (21.1%) (**Figure 2**).

## 4 Discussion

We found that the most frequent isolates were *S. aureus, E. coli, P. aeruginosa, P. vulgaris, and M. morganii.* These bacteria showed a higher common resistance to Ampicillin, Ciprofloxacin, Levofloxacin, Trimethoprim-sulfamethoxazole, and Cefuroxime. It is noteworthy that E. coli had the highest resistance to antibiotics, while Gentamicin, Tobramycin, and Levofloxacin were the most effective antibiotics against the most prevalent bacteria. Notably, there was a significant sensitivity to Meropenem, Piperacillin/tazobactam, and Amikacin among Gram-negative bacteria.

The study’s strengths include the use of an updated database compared to other national studies between 2010 and 2016 (9, 15) and the use of automated methods for analyzing antibiotic resistance. Additionally, the findings contribute scientifically to Spanish-speaking countries, as often the results align with foreign studies but are not mentioned or included in them (16–21).

Our results indicate that although Gram-negative bacteria were the most common (63%), Staphylococcus aureus was the most frequently isolated species. Similar results were found in China (17.7%) (17), Nigeria (15.6%) (18), and Sudan (18.2%) (19). Together, these results agree with an international microbiological review, where *S. aureus* remains one of the most important pathogens in diabetic foot infections, with a frequency of approximately 50% in monomicrobial infections. Additionally, the incidence of *P. aeruginosa* is increasing, ranging from 10% to 26.6% (15). Although this pathogen was not the most frequent in this study, studies conducted in Nicaragua found a prevalence of 24.4% and 38.8% (22, 23).

At the Hospital Nacional Edgardo Rebagliati Martins of EsSalud, Gram-negative bacteria predominated (69.5%). However, regarding the frequency of bacteria, the results were opposite to ours, with E. coli being the most common bacteria (23.4%), followed by *E. faecalis* (14.1%) and S. aureus (13.3%) (9). Similar findings were seen in a provincial hospital, where 64.29% of the bacteria were Gram-negative, with E. coli being the most frequent (16.07%), followed by S. aureus (14.29%) (15). Foreign countries also showed similar results, with *E. coli* being the most frequent bacteria in Iran with 20.5% (20), and Lebanon with 15% (21). Considering these reports, *S. aureus, E. coli, and P. aeruginosa* appear to be the most prevalent bacteria in diabetic foot infections worldwide.

Regarding the antibiotic susceptibility pattern of the bacteria, it was observed that Gram-negative bacteria showed high levels of antimicrobial resistance to Ampicillin (89.7%), Cefuroxime (75.9%), Trimethoprim-sulfamethoxazole (64.6%), and Ciprofloxacin (61.5%). On the other hand, they displayed higher sensitivity to Carbapenems (>85.4%), Amikacin (85.3%), and Piperacillin/tazobactam (82.5%), while Ampicillin (6.9%) and Fosfomycin (1.5%) had lower sensitivity. These findings differ from another Peruvian study, where P. aeruginosa and A. baumannii showed a high resistance to carbapenems of 83% and 100%, respectively (9). However, both studies agree that Gram-negative bacteria showed low sensitivity to Ampicillin and high sensitivity to Carbapenems and Amikacin (15). Additionally, Enterobacteria (*E. coli, K. pneumoniae, P. mirabilis, and P. vulgaris*) showed a resistance rate of 89.4% to ciprofloxacin, which is a first-line drug in treatment (9). In Nigeria, Gram-negative bacteria also displayed high resistance to Trimethoprim-sulfamethoxazole (89%) and ciprofloxacin (54.3%) (18).

In Gram-positive bacteria, a higher antimicrobial resistance was observed to Penicillin (95.4%), Ampicillin (87.7%), Clindamycin (80.7%), and Oxacillin (76.4%). However, a 100% antimicrobial sensitivity was found for Daptomycin, Vancomycin, and Teicoplanin. These results align with other Peruvian studies where a 71% resistance to Oxacillin in these bacteria was found (9, 15). In Nigeria, resistance to Penicillin G was also observed (66.1%), along with low resistance to Piperacillin/tazobactam (6.8%) and Amikacin (10.2%) (18).

Focusing on bacteria with the highest incidence, especially S. aureus, internationally, high levels of antibiotic resistance have been observed, representing a significant risk and limiting future treatment options. Specifically, Methicillin resistance rates range from 16% to 44%, even reaching 50% in Lebanon (16, 21). In this study, bacteria with the highest incidence, such as *S. aureus, E. coli, P. aeruginosa, Proteus vulgaris, and Morganella morganii*, showed a high percentage of common resistance to Ampicillin, Ciprofloxacin, Levofloxacin, Trimethoprim-sulfamethoxazole, and Cefuroxime. Additionally, other studies have found that Pseudomonas aeruginosa is resistant to any carbapenem, with a prevalence ranging from 5.4% (16).

As previously mentioned, Gentamicin, Tobramycin, and Levofloxacin showed the highest sensitivity to the most incident bacteria. In China, Gram-positive bacteria showed low resistance to Gentamicin (17), whereas in Nigeria, both Gram-positive (40.1%) and Gram-negative (54.3%) bacteria displayed high resistance to this antibiotic (18). Sudan recorded a resistance rate of 65.2% for *S. aureus*, while Nicaragua observed complete resistance to this bacterium (23).

The results obtained reveal variability in bacterial susceptibility to antibiotics, but most of them show concerning resistance to these medications. A recent systematic review demonstrated that infections caused by multidrug-resistant bacteria have increased in recent years, associated with a higher prevalence of diabetic foot ulcers (24). This phenomenon is linked to the prolonged use of broad-spectrum antibiotics, necessary to penetrate the bacterial biofilm but also triggering survival mechanisms and increased resistance. This has a negative impact on amputation and mortality rates in diabetic patients (24). It is crucial to address this problem and seek effective therapeutic alternatives to fight infections in diabetic foot, avoiding the indiscriminate use of antibiotics and promoting strategies that limit the development of bacterial resistance.

On the other hand, sodium-glucose cotransporter-2 (SGLT-2) inhibitors have emerged as a valuable addition to the therapeutic arsenal for managing diabetes mellitus. These medications function by promoting glycosuria, leading to reduced blood glucose levels. While this effect can be beneficial for glycemic control, it may inadvertently contribute to impaired tissue perfusion in the lower extremities, which is a well-established risk factor for diabetic foot complications (25). As such, there is a growing need for comprehensive research to elucidate the precise relationship between SGLT-2 inhibitors and diabetic foot infections, shedding light on the clinical implications and guiding the development of preventive strategies in diabetic patient populations.

Another contemporary aspect is the role of SARS-CoV-2 infections on diabetic foot. The COVID-19 pandemic has had far-reaching effects on global healthcare systems and has also influenced the management and severity of diabetic foot syndrome (26). Individuals with diabetes are already predisposed to various complications, including this syndrome, due to factors such as neuropathy and impaired vascular function. However, the pandemic has introduced several additional challenges that have the potential to exacerbate the severity of diabetic foot (27). Firstly, disruptions in healthcare access and routine check-ups during lockdowns or overwhelmed healthcare systems have made it difficult for diabetic patients to receive timely foot care and monitor their condition (28). Secondly, some studies have shown that individuals with poorly controlled diabetes are at higher risk of severe COVID-19 outcomes, which may indirectly worsen diabetic foot severity by affecting overall health and immune responses (29, 30). Furthermore, the stress and anxiety associated with the pandemic have led to lifestyle changes, including altered dietary habits and reduced physical activity, which can further contribute to poor glycemic control and increased diabetic food syndrome risk (31).

## 5 Limitation

This study has certain limitations that need to be considered when interpreting the results. Firstly, the sample was limited to diabetic foot patients from a single tertiary hospital, which implies that the frequency of bacteria, as well as their resistance and sensitivity to antibiotics, may vary in other health centers located in urban, rural, or mountainous areas (15). Therefore, caution is necessary when generalizing the findings to other populations and clinical settings. Another important limitation is that the study did not consider the presence of fungi, such as *Candida albicans and/or Candida tropicalis*, which often coexist and have fungal growth alongside the studied bacteria (32). The omission of these microorganisms could have affected the complete understanding of infections associated with diabetic foot and their treatment. Despite these limitations, this study has successfully identified the most common bacteria in diabetic foot infections and their resistance and sensitivity profiles to different antibiotics used in clinical practice. These findings provide valuable information for the management and treatment of infections in patients with diabetic foot, although a broader and more comprehensive evaluation in future studies is required to address the mentioned limitations and obtain a more accurate view of the situation.

## 6 Conclusion and future direction

In this study, S. aureus, E. coli, P. aeruginosa, P. vulgaris, and M. morganii were identified as the most common bacteria in diabetic foot infections. These bacteria showed resistance to various antibiotics, such as Ampicillin, Ciprofloxacin, Levofloxacin, Trimethoprim-sulfamethoxazole, and Cefuroxime, and Escherichia coli was the most resistant. However, Levofloxacin was found to be one of the most effective antibiotics against these prevalent bacteria. Additionally, Gram-negative bacteria showed notable sensitivity to Meropenem, Piperacillin/tazobactam, and Amikacin.

Bacterial resistance in diabetic foot infections is a growing global concern as it hinders treatment and increases the risk of serious complications such as the need for amputation and mortality. Therefore, it is crucial to focus on studying the susceptibility of bacteria to antibiotics to ensure appropriate prescription dosages in pharmacological treatment and to avoid overuse of these medications. Understanding the resistance and sensitivity of bacteria causing diabetic foot infections is essential to guide the choice of antibiotics and ensure effective treatment. Furthermore, it would be pertinent to consider the assessment of fungal infections in future research endeavors. This inclusion would contribute to a more comprehensive understanding of the microbiota implicated in these infections, further enriching the scope of the study. This will help optimize clinical outcomes and reduce complications associated with these infections. Continued research and data updates on bacterial resistance are necessary to adapt therapeutic strategies and effectively address this public health challenge.

## Data availability statement

The original contributions presented in the study are included in the article/supplementary material. Further inquiries can be directed to the corresponding author.

## Ethics statement

The studies involving humans were approved by Ethics Committee of the Hospital María Auxiliadora (HMA/CIEI/008/2021, May 26, 2021) and the Universidad Norbert Wiener (Exp.528-2021, April 26, 2021). The studies were conducted in accordance with the local legislation and institutional requirements. The participants provided their written informed consent to participate in this study.

## Author contributions

JM-S: Conceptualization, Formal analysis, Methodology, Project administration, Supervision, Writing – original draft, Writing – review & editing, Investigation, Visualization. JC: Conceptualization, Data curation, Investigation, Writing – original draft. DP-R: Conceptualization, Data curation, Investigation, Writing – original draft, Visualization. EG-P: Data curation, Formal analysis, Writing – review & editing, Visualization. CS: Investigation, Methodology, Writing – review & editing, Conceptualization, Validation. HC-P: Methodology, Writing – review & editing, Project administration, Supervision.
